# Response of the nosZ-type denitrifying microbial community and metabolic characteristics to precipitation changes in the alpine wetland

**DOI:** 10.3389/fmicb.2025.1581432

**Published:** 2025-04-24

**Authors:** Ni Zhang, Hongchen Jiang, Zhiyun Zhou, Yijun Wang, Desheng Qi, Shijia Zhou, Jing Ma, Kelong Chen

**Affiliations:** ^1^Qinghai Province Key Laboratory of Physical Geography and Environmental Process, College of Geographical Science, Qinghai Normal University, Xining, China; ^2^Key Laboratory of Tibetan Plateau Land Surface Processes and Ecological Conservation (Ministry of Education), Qinghai Normal University, Xining, China; ^3^National Positioning Observation and Research Station of Qinghai Lake Wetland Ecosystem in Qinghai, National Forestry and Grassland Administration, Hubei, Haibei, China; ^4^State Key Laboratory of Biogeology and Environmental Geology, China University of Geosciences, Wuhan, China; ^5^Qinghai Provincial Key Laboratory of Geology and Environment of Salt Lakes, Qinghai Institute of Salt Lakes, Chinese Academy of Sciences, Xining, China

**Keywords:** Qinghai-Tibetan Plateau, climate change, extreme precipitation, carbon and nitrogen cycling, LC/MS

## Abstract

The impact of climate change on the global hydrological cycle is becoming increasingly significant, with changes in precipitation patterns emerging as a key factor influencing the carbon and nitrogen cycling processes in alpine wetland ecosystems. However, the response of the nosZ-type denitrifying microbial community and its metabolic characteristics in the source wetland to precipitation changes remains unclear. In this study, high-throughput sequencing of the nosZ gene and LC-MS-based metabolomics were used to investigate the response of the nosZ-type denitrifying microbial community and its metabolic characteristics to precipitation changes (4 years) in the source wetland of Qinghai Lake. The results showed that *Microvirga* was the key bacterial group in the source wetland of Qinghai Lake, and *Azospirillum* was sensitive to changes in precipitation (*P* < 0.05). The 50% rainfall enhancement treatment significantly increased soil moisture, and the total carbon content showed an increasing trend with the increase in precipitation (*P* < 0.05). pH was the most important explanatory factor for community structure, while total nitrogen content was the key explanatory factor for community diversity. Deterministic processes dominated the assemblage of the nosZ-type denitrifying microbial community in the source wetland of Qinghai Lake. Soil metabolomics analysis showed that the differential metabolites in the Source Wetland mostly exhibited significant positive correlations. Precipitation changes significantly affected the relative abundance of N-Acetylaspartic acid. In summary, lower precipitation is more favorable for maintaining carbon storage in the source wetlands of Qinghai Lake. Precipitation variation disrupted the existing nitrogen balance within the ecosystem and altered the structure of the nosZ-type denitrifying microbial community and soil metabolic characteristics. These findings imply that climate change-driven shifts in precipitation patterns may impact carbon and nitrogen dynamics in alpine wetlands, alter ecosystem stability, and have profound effects on microbial communities and biogeochemical cycles.

## Introduction

Wetland ecosystems, integral components of biogeochemical cycles, play pivotal roles in climate regulation, water purification, and the preservation of biodiversity (Asanopoulos et al., 2021; Temmink et al., 2022). Denitrification, a complex series of microbially-mediated metabolic processes, facilitates the conversion of nitrate into nitrogen gas, which is subsequently released into the atmosphere (Shoun and Tanimoto, 1991; Domeignoz-Horta et al., 2016). This process is critically important for the nitrogen cycle within wetland environments. The presence of the N_2_O reductant, which serves as the sole sink for nitrous oxide (N_2_O), significantly influences the dynamic source-sink interactions of N_2_O in these ecosystems. To date, only a singular biological pathway for N_2_O reduction has been elucidated, wherein the nitrous oxide reductase gene (*nosZ*) catalyzes the conversion of N_2_O to nitrogen gas (N_2_) (Hallin et al., 2018). Consequently, investigating the microbial community structure that harbors the *nosZ* gene, along with its environmental determinants, is essential for mitigating nitrous oxide emissions and alleviating the greenhouse effect (Shan et al., 2021). Furthermore, the efficacy of the denitrification process is intricately linked to the hydrological contexts of wetland habitats, particularly the ramifications of precipitation fluctuations on water levels, soil moisture, and redox conditions, all of which can profoundly affect the composition and functionality of denitrifying microbial communities (Shi et al., 2021; Zhang et al., 2024).

In recent years, as the ramifications of climate change on the global hydrological cycle have become increasingly pronounced, alterations in precipitation patterns have emerged as a pivotal factor influencing the functioning of wetland ecosystems. The distinctive characteristics of alpine regions render the ecological functions and nutrient cycling within these wetlands particularly susceptible to fluctuations in precipitation (Han et al., 2021). This phenomenon is especially evident within the Tibetan Plateau, where seasonal variations in precipitation and the increasing frequency of extreme climatic events may exert intricate impacts on the microbial communities and their metabolic activities in alpine wetlands (Gao et al., 2014; Jansson and Hofmockel, 2020). Studies conducted in other contexts have demonstrated that shifts in precipitation can modify the composition and activity of denitrifying microbial communities by regulating critical factors such as wetland water levels, oxygen availability, and organic matter input (Zhang et al., 2024; Wang J. et al., 2018; Zou et al., 2019). However, the processes of denitrification display considerable spatiotemporal variability across diverse environmental contexts (Liu et al., 2003; Wang et al., 2014), and different wetland types manifest distinct characteristics in terms of nitrous oxide emissions. For instance, marsh wetlands are typically recognized as substantial sources of nitrous oxide (Wang X. et al., 2018), whereas riverine wetlands may function as sinks for this greenhouse gas (Zhang et al., 2021). Although some research has been conducted on nosZ-type denitrifying microorganisms, the findings are often disparate. For example, Lin et al. (2023) investigated the dynamics of nosZ-type denitrifying microorganisms within coastal wetlands and observed that their abundance diminished with increasing soil depth, while exhibiting a positive correlation with salinity, total carbon, and total nitrogen. Conversely, Hu et al. (2023) explored the denitrifying microorganisms inhabiting estuarine wetlands, revealing a positive correlation between nosZ-type denitrifying microorganisms and both salinity and redox potential in the downstream sections of the Pearl River Estuary. Nevertheless, the precise effects of changes in precipitation on the community structure and metabolic profiles of nosZ-type denitrifying microorganisms in alpine source wetlands remain inadequately understood.

The Qinghai Lake Basin, situated in the northeastern region of the Qinghai-Tibet Plateau, is recognized as a vital high-latitude international wetland and serves as a critical indicator zone for climate change impacts on the Qinghai-Tibet Plateau (Tang et al., 1992; Cui and Li, 2015). As the primary zone for wetland distribution on the Tibetan Plateau, this area’s climate change displays notable heterogeneity. Compared to other land surfaces, the Qinghai Lake wetland possesses unique ecological characteristics (Li et al., 2019; Qi, 2012). Consequently, we have chosen the Qinghai Lake alpine wetland as the focal point for our research and simulated precipitation changes by implementing various precipitation gradients. This study aims to address the following questions: (1) to systematically analyze the response patterns of the nosZ-type denitrifying microbial community and its metabolic characteristics to changes in precipitation in the source wetland; (2) to investigate the impact of precipitation-driven changes in soil physicochemical properties on the nosZ-type denitrifying microbial community and soil metabolites; (3) to analyze the changes in the process of nosZ-type denitrifying microbial community assembly under different precipitation gradients. By thoroughly examining the denitrifying microbial communities and soil metabolic characteristics, we aim to reveal the potential impacts of precipitation changes on the source wetland ecosystem, thereby providing crucial theoretical insights into how wetland ecosystems respond to climate change. This research will also offer scientific support for the development of effective wetland conservation and management strategies.

## Materials and methods

### Overview of the sampling sites

The Wayan Mountain Experimental Station (37°43′–37°46′N, 100°01′–100°05′E) is located at the source of the Shaliu River on the northern shore of Qinghai Lake, Qinghai Province, China, with an elevation ranging from 3,720 to 3,850 m. The climate is characterized by large diurnal temperature variation, with the highest average daily temperature typically in July and the lowest in January. The multi-year average temperature is −3.31°C, and the multi-year average annual precipitation is 420.37 mm (Zhang et al., 2024). The vegetation and soil types in the area are simple, with dwarf grasses as the dominant vegetation and meadow soils as the primary soil type (Zhang et al., 2024).

### Sample collection

The plot was established in 2018, and the actual situation of the sample land is shown in Supplementary Figure 1. We reduced the natural precipitation by installing equidistant inclined diversion channels of different areas. The diversion channels collect rainwater into the horizontal tank in the concourse, and increase the natural precipitation through the spray device. In 15 June 2022 (early in the growing season), soil samples were collected from the 0 to 10 cm surface layer using a 4.5 cm diameter soil auger in an “S”-shaped pattern, following a five-point sampling method. A total of 15 soil samples were collected, with 5 treatments × 3 replicates, and the samples were labeled according to the treatment groups as follows: C (natural control), + 50% (50% rainfall increase), + 25% (25% rainfall increase), −50% (50% rainfall reduction), and −25% (25% rainfall reduction). All samples were sieved through a 2 mm mesh to remove visible stones and plant debris, then immediately stored in ice bags and transported to the laboratory. Some soil samples were stored at −80°C for DNA extraction, while others were used for the analysis of soil physicochemical properties.

### Determination of soil biogeochemical properties

Soil pH was measured using a pH probe (FE20-FiveEasy pH, Mettler Toledo, Germany) at a fresh soil-to-water ratio of 1:25. Total carbon (TC) and total nitrogen (TN) were determined using an elemental analyzer (Vario EL III, Elemental Analysis System GmbH, Germany). Soil moisture content (Hum) and temperature (Tmp) at a depth of 0-10 cm were monitored using a TDR-300 soil moisture probe (Spectrum Technologies Inc., Plainfield, Illinois, United States) and a LI-8100 sensor (LI-COR Inc., Lincoln, Nebraska, United States) (Zhang et al., 2023).

### DNA extraction and polymerase chain reaction

To minimize PCR bias, each sample was analyzed in triplicate for microbial experiments. DNA was extracted using the PowerSoil DNA Isolation Kit (MoBio Laboratories, Carlsbad, CA, United States) according to the kit protocol, and DNA concentration and purity were measured using a NanoDrop2000 UV-Vis spectrophotometer (Thermo Scientific, Waltham, MA, United States). The *nosZ* gene was amplified using primers nosZ-F (5′-CGYTGTTCMTCGACAGCCAG-3′) and nosZ-1622R (5′-CGSACCTTSTTGCCSTYGCG-3′) (Throbäck et al., 2004). The PCR amplification process followed the method described by Wang et al. (2024) Amplicons were purified using the PowerClean DNA Kit (MoBio Laboratories, Carlsbad, CA, United States) and paired-end sequencing was performed on 15 samples on the Illumina MiSeq platform (San Diego, United States). The raw sequences were processed using Cutadapt (v. 1.9.1) (Martin, 2011) to identify and remove primer sequences, followed by assembly and filtering with Usearch (v. 10.0) (Edgar, 2018). The denoising algorithm (DADA2) within the QIIME2 pipeline^1^ was then applied, including denoising, merging, and non-chimeric sequence identification, to define amplicon sequence variants (ASVs) (Callahan et al., 2016). Finally, with the localization of the NT (2019.8 download)^2^ database, for each representative ASV sequence classification and recognition. This part of the work was completed by Shanghai Ling En Biological Co., Ltd.

### Non-targeted metabolism

To monitor the stability of the analysis, quality control (QC) samples were injected during the metabolomics analysis, which were prepared by mixing equal amounts of all the samples. The extracts were analyzed using reverse-phase ultrahigh-performance liquid chromatography (RP-UHPLC, Shimadzu Handels GmbH, Köln, Germany) coupled online with electrospray ionization quadrupole time-of-flight mass spectrometry (QqTOF-MS). The mobile phases consisted of 3 mmol/L ammonium formate (elution solvent A) and acetonitrile (elution solvent B) (Ware et al., 2024). The gradient elution program followed the method described by Ware et al. (2024) The chromatographic eluate was introduced into the Sciex TripleTOF 6600 LC-MS system (AB Sciex, Darmstadt, Germany) through the DuoSpray™ ion source’s ESI part, operating in SWATH (Sequential Window Acquisition of All Theoretical Fragment Ion Spectra) mode to collect data in all positive ion scanning windows, and controlled by Analyst™TF software 1.8 (Ware et al., 2024).

### Statistical analysis

Alpha diversity indices (Chao1, ACE, Simpson, and Shannon indices) were calculated using the “get_alphaindex” function from the MicrobiotaProcess package (version 1.18.0) in R software (version 4.1.2), and rarefaction curves were plotted using the “ggrarecurve” function. Principal Component Analysis (PCA) was performed using the “pca” function from the PCAtools package (version 2.18.0), and Partial Least Squares Discriminant Analysis (PLS-DA) was conducted using the “plsda” function from the mixOmics package (version 6.30.0). Microbial functional groups were predicted using FAPROTAX (Liang et al., 2020). Data correlations were computed using the “mantel_test” function from the linkET package (version 0.0.7.4), and correlation network plots were generated. Variable Importance in Projection (VIP) scores for group comparisons were calculated using the “opls” function from the ropls package (version 1.38.0). A distance matrix based on Bray-Curtis dissimilarity was calculated to assess species dissimilarity. Phylogenetic trees were pruned using the “prune.sample” function from the Picante package (version 1.8.2). The beta Nearest Taxon Index (betaNTI) was computed using the “pNST” function from the NST package (version 3.1.10) (rand = 1,000, nworker = 4), and the Raup-Crick index (RCbray) was calculated using the microeco package (version 1.11.0) (runs = 1,000) to determine the relative importance of deterministic and stochastic processes in community assembly. Differences in the data were analyzed using the Kruskal-Wallis test (Dunn’s Test), and all other analyses and visualizations were performed using the ggplot2 package (version 3.5.1) in R software (version 4.1.2).

## Results

### Response of environmental factors to precipitation changes

**FIGURE 1:**
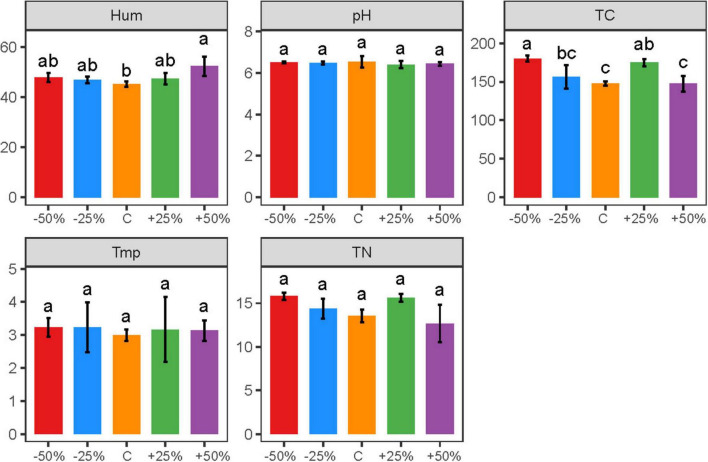
Changes in the content of physicochemical factors caused by precipitation changes. Letters a-d indicate significance, with the same letter indicating no significant difference (*p* > 0.05); Tmp, soil temperature; Hum, soil humidity; TN, total nitrogen; TC, total carbon; pH, soil pH.

Precipitation changes caused significant differences in soil moisture and total carbon content in the treatments (Figure 1, *P* < 0.05). Soil moisture ranged from 44.10 to 56.20%, with a significant increase in soil moisture under the 50% increased precipitation treatment. The 50% reduction and 25% increase in precipitation significantly increased the total carbon content, while the total carbon content in other treatments was slightly higher than the natural control, but the difference was not statistically significant (Figure 1). The trends in total nitrogen content were consistent with those of total carbon content, but the changes in total nitrogen content were not statistically significant (Figure 1, *P* > 0.05). Furthermore, soil pH and temperature were less affected by precipitation changes (Figure 1, *P* > 0.05).

### Response of the nosZ-type denitrifying microbial sequence to changes in precipitation

**FIGURE 2:**
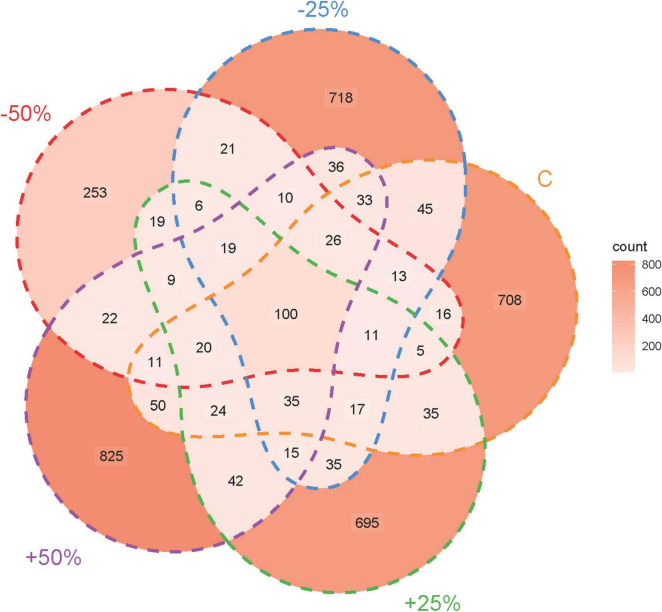
Sequencing characteristics of the nosZ-type denitrifying microbial community in treatments under changes in precipitation.

The rarefaction curve confirmed the adequacy of the sequencing data volume (Supplementary Figure 2). Sequencing results of denitrifying microorganisms in treatments showed that the number of sequences obtained for the nosZ-type denitrifying microbial community ranged from 332,670 to 410,500. Precipitation changes led to divergent ASV numbers among the groups, with only 100 ASVs shared across all groups. The total number of ASVs for −50%, −25%, C, + 25%, and + 50% were 560, 1,139, 1,148, 1,086, and 1,277, respectively, with unique ASV counts of 253, 718, 708, 695, and 825 (Figure 2).

### Response of the diversity of nosZ-type denitrifying microbial communities to precipitation changes

**FIGURE 3:**
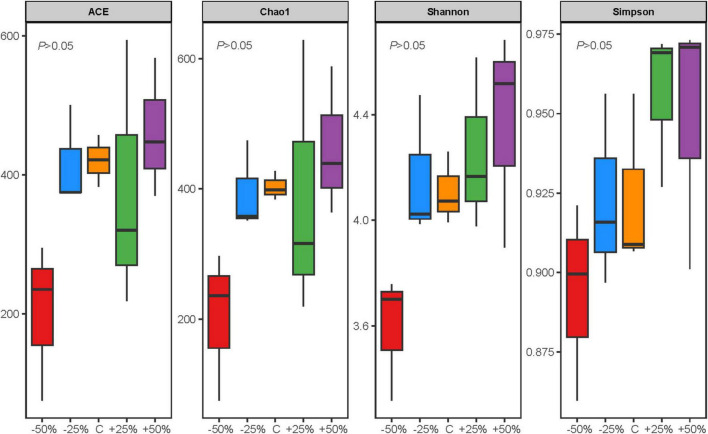
Alpha diversity indices of the nosZ-type denitrifying microbial community in treatments under changes in precipitation. There is no statistically significant difference between groups (*p* > 0.05).

Precipitation changes did not significantly affect the Alpha diversity of nosZ-type denitrifying microorganisms in the evaluated soil samples (*P* > 0.05). However, there were some differences in the response patterns of denitrifying microbial richness and evenness to different precipitation gradients (Figure 3). The species richness (ACE and Chao1 indices) and evenness (Shannon and Simpson indices) of the nosZ-type denitrifying microorganisms generally showed a gradual increase with increasing precipitation (Figure 3). Principal Component Analysis (PCA) of the data (Supplementary Figure 3) revealed that the first axis explained 63.23% of the variance and the second axis explained 9.58%, with a total explained variance exceeding 70%, suggesting that precipitation changes contributed to the differences in the nosZ-type denitrifying microbial community of treatments (Supplementary Figure 3).

### Response of the nosZ-type denitrifying microbial community structure to precipitation changes

**FIGURE 4:**
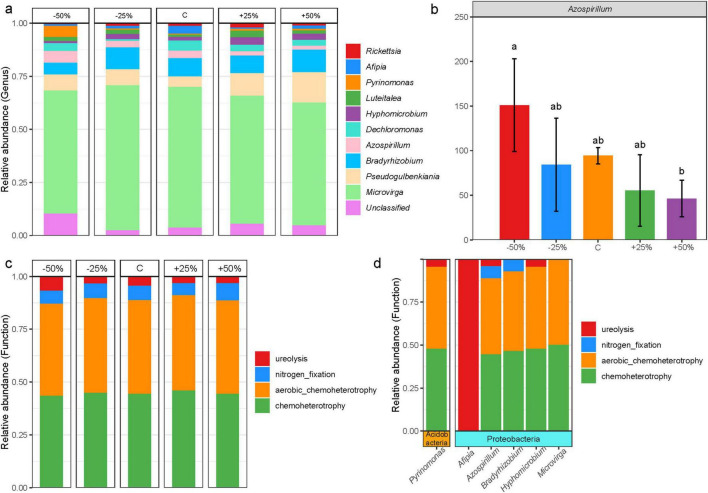
Composition of the nosZ-type denitrifying microbial community in treatments under changes in precipitation; **(a)** dominant genera in treatments; **(b)** differential genera in treatments; **(c)** predicted major functional groups of denitrifying microorganisms; **(d)** microbial groups corresponding to the predicted major functional groups. Letters ab in **(b)** indicate significance, with the same letter indicating no significant difference (*p* > 0.05) and different letters indicating significant differences (*p* < 0.05).

The dominant phyla of nosZ-type denitrifying microorganisms in treatments (Supplementary Figure 4) were Proteobacteria (89.54%) and Actinobacteria (3.77%), which together accounted for more than 93% of the total sequences. The response to precipitation changes was not significant (*P* > 0.05). The dominant microbial genera were *Microvirga* (56.70%), *Pseudogulbenkiania* (8.19%), *Bradyrhizobium* (7.89%), *Azospirillum* (2.93%), *Dechloromonas* (2.77%), *Hyphomicrobium* (2.09%), *Luteitalea* (1.74%), *Pyrinomonas* (1.48%), *Afipia* (1.43%), and *Rickettsia* (1.15%), which together accounted for more than 86% of the total sequences (Figure 4a). *Azospirillum* was the only genus with a significant response to precipitation changes (Figure 4b, *P* < 0.05), with its relative abundance exhibiting an inverse trend to that of precipitation. Compared to a 50% reduction in precipitation, a 50% increase in precipitation significantly reduced the relative abundance of this genus (Figure 4b).

FAPROTAX functional predictions revealed that the ecological functions of the nosZ-type denitrifying microbial community in treatments were classified into four predicted functional groups (Figure 4c, relative abundance > 1%), namely, chemoheterotrophy (39.05%), aerobic chemoheterotrophy (38.83%), nitrogen fixation (5.94%), and ureolysis (3.65%), which together accounted for more than 87% of the total. Precipitation changes appear to had little effect on the major functional groups of nosZ-type denitrifying microorganisms (*P* > 0.05). The four functional groups were further linked to denitrification-dominant genera, revealing that six genus-level microbial groups from two phyla were mainly involved in these processes (Figure 4d). The majority of the microbial groups were closely associated with the processes of aerobic chemoheterotrophy and chemoheterotrophy, while *Afipia* was involved exclusively in ureolysis (Figure 4d). Additionally, *Azospirillum* and *Bradyrhizobium* were the primary microbial groups involved in nitrogen fixation (Figure 4d).

### Correlation analysis of soil physicochemical properties and nosZ denitrification microorganisms

**FIGURE 5:**
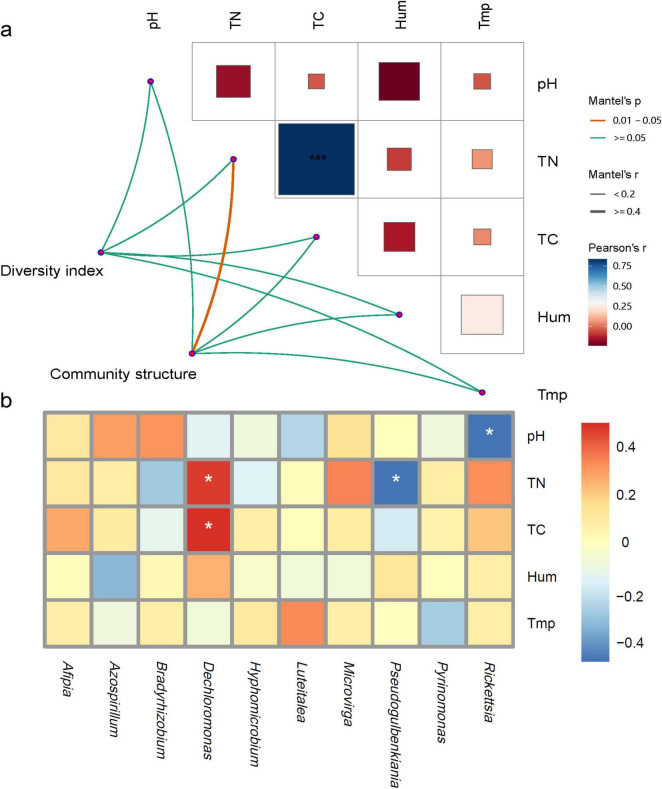
Correlation analysis of soil physicochemical properties and nosZ denitrification microorganisms; **(a)** correlation network of denitrifying microbial community characteristics with physicochemical factors; **(b)** correlation heatmap of dominant genera and physicochemical factors. *Indicates *p* < 0.05, ***indicates *p* < 0.001; Tmp, soil temperature; Hum, soil humidity; TN, total nitrogen; TC, total carbon; pH, soil pH.

Mantel correlation analysis (Figure 5a) revealed that the Alpha diversity of the nosZ-type denitrifying microorganisms was minimally influenced by environmental factors, while the dominant microbial groups were significantly correlated with total carbon content. All environmental factors showed positive correlations, with total carbon and total nitrogen being significantly positively correlated (*P* < 0.05). To further refine the relationship between environmental factors and denitrifying microbial communities, a correlation heatmap was constructed. The results showed that *Dechloromonas* was significantly positively correlated with total carbon and total nitrogen, *Pseudogulbenkiania* was significantly negatively correlated with total nitrogen, and Rickettsia was significantly negatively correlated with pH (Figure 5b, *P* < 0.05).

### Response of nosZ-type denitrifying microbial community structure to precipitation changes

**FIGURE 6:**
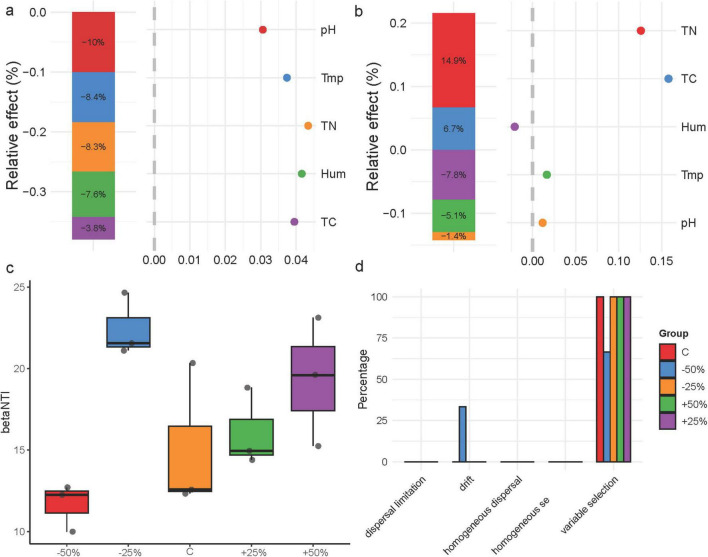
Factors influencing the nosZ-type denitrifying microbial community and community construction in treatments; **(a)** hierarchical partitioning analysis of factors affecting denitrifying microbial community structure; **(b)** hierarchical partitioning analysis of factors affecting denitrifying microbial community diversity; **(c)** distribution of βNTI index; **(d)** distribution of denitrifying microbial community construction processes. Tmp, soil temperature; Hum, soil humidity; TN, total nitrogen; TC, total carbon; pH, soil pH.

To analyze the specific effects of environmental factors on the nosZ-type denitrifying microbial community in treatments, generalized linear models were used for hierarchical partitioning analysis (Figures 6a,b). The results showed that environmental factors negatively affected the nosZ-type denitrifying microbial community structure, with pH being the most important explanatory factor for community structure, accounting for 10% of the variation (Figure 6a). Total nitrogen and total carbon had a positive effect on community diversity, with total nitrogen being the most important explanatory factor for diversity, accounting for 14.9% of the variation (Figure 6b). The βNTI values for the nosZ-type denitrifying microbial community in treatments showed a generally increasing trend with increasing precipitation (*P* > 0.05), indicating that deterministic processes predominated in shaping the denitrifying microbial community structure in treatments (|βNTI| > 2) (Figure 6c). Further calculation of RCbray revealed the relative effects of diffusion limitation, drift, homogeneous diffusion, and selection on the dynamics of the denitrifying microbial community (Figure 6d). The results showed that precipitation changes did not alter the overall construction process of the nosZ-type denitrifying microbial community in treatments; however, the relative contributions of deterministic and stochastic processes varied under different precipitation treatments. Heterogeneous selection dominated the formation of the denitrifying microbial community, with a 100% contribution in −25%, C, + 50%, and + 25% treatments, and 66.6% in −50% (Figure 6d). Additionally, there was a certain drift effect in the community construction under the −50% treatment, accounting for 33.3% (Figure 6d).

### Response of soil metabolites to precipitation changes

**FIGURE 7:**
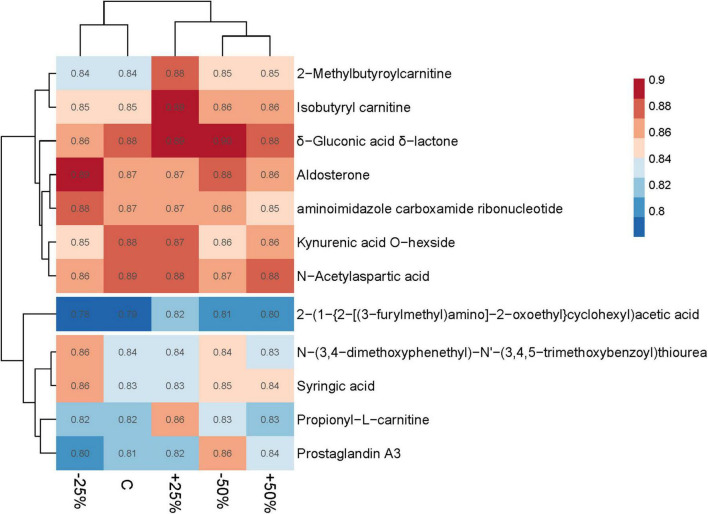
Heatmap of different metabolites under precipitation changes in treatments.

PCA (Principal Component Analysis) revealed differences in metabolic profiles among groups following precipitation changes, with a total explained variance of 94.51% (Supplementary Figure 5). Precipitation changes significantly affected the relative abundance of 12 metabolites (VIP ≥ 1). Except for six metabolites of unknown categories, the relative abundance of two esterified fatty acid metabolites initially decreased and then increased with increasing precipitation, while the relative abundance of metabolites from the other four subcategories initially increased and then decreased with increasing precipitation (Figure 7).

### The correlation analysis of soil metabolites with environmental factors and the dominant denitrifying microbial communities

**FIGURE 8:**
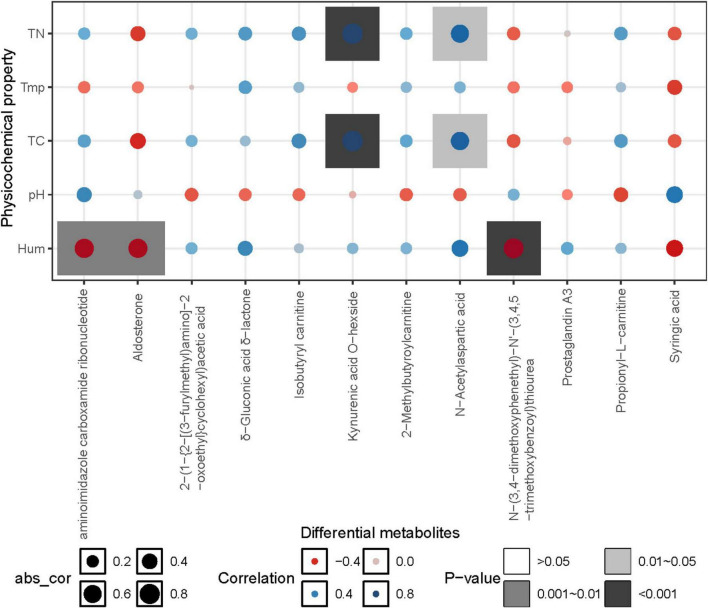
Correlation heatmap between metabolites and environmental factors. Tmp, soil temperature; Hum, soil humidity; TN, total nitrogen; TC, total carbon; pH, soil pH.

The correlation heatmap illustrated the relationship between differential metabolites and environmental factors. The results showed that differential metabolites were mainly influenced by carbon and nitrogen content, as well as soil moisture. Specifically, the relative abundances of Kynurenic acid O-hexside and N-Acetylaspartic acid exhibited a significant positive correlation with total carbon (TC) and total nitrogen (TN) (*p* < 0.05). On the other hand, aminoimidazole carboxamide ribonucleotide, Aldosterone, and N-(3,4-dimethoxyphenethyl)-N’-(3,4,5-trimethoxybenzoyl) thiourea showed a highly significant negative correlation with soil moisture (*p* < 0.01) (Figure 8).

**FIGURE 9:**
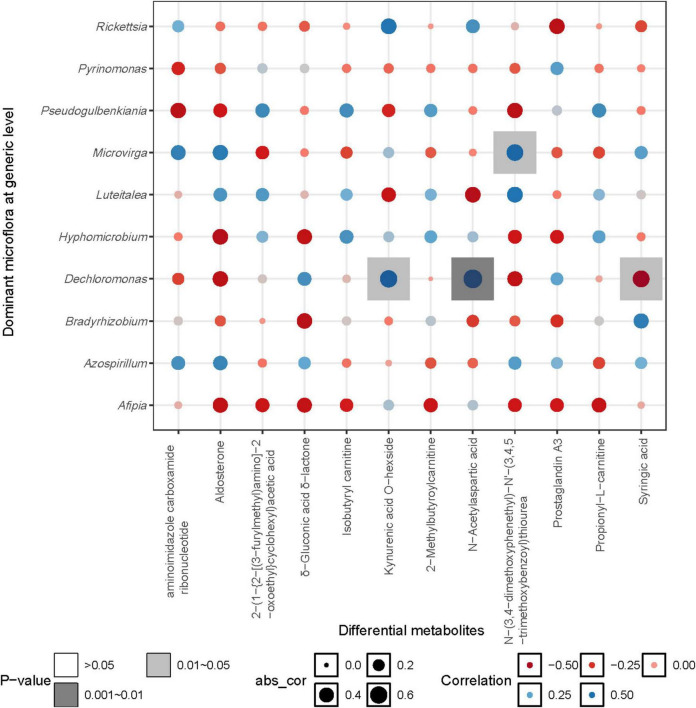
Correlation heatmap between metabolites and denitrifying microorganisms.

The correlation heatmap between the dominant bacterial genera (relative abundance > 0.01) and differential metabolites (relative abundance > 0.001, *p* < 0.05) showed that N-(3,4-dimethoxyphenethyl)-N’-(3,4,5-trimethoxybenzoyl) thiourea was significantly positively correlated with *Microvirga*, while Kynurenic acid O-hexside and N-Acetylaspartic acid were significantly positively correlated with *Dechloromonas*. Additionally, Syringic acid was significantly negatively correlated with *Dechloromonas* (*p* < 0.05) (Figure 9).

## Discussion

### Response of the nosZ-type denitrifying microbial community diversity and composition to precipitation changes

Alterations in precipitation patterns can substantially influence soil microbial communities by modifying soil moisture content, which consequently impacts ecosystem functions (Nielsen and Ball, 2015; Ochoa-Hueso et al., 2020). In the present study, we observed that fluctuations in precipitation did not significantly affect the alpha diversity of the nosZ-type denitrifying microbial community across the various treatments. However, community diversity exhibited a positive correlation with changes in precipitation. This suggests that an increase in precipitation may likely exacerbate N_2_O emissions (Maeda et al., 2010; Zhang et al., 2015). Uchida et al. (2014) investigated the response of denitrifying genes to waterlogging events and found that elevated soil moisture levels significantly enhanced the abundance of soil denitrifying bacteria. Additionally, research conducted by Chen et al. (2019) concluded that nosZ-type denitrifying microorganisms maintained relative stability in soil across varying environmental conditions, with diversity assessments generally revealing no significant differences. These findings reinforce the results of the current study.

The phylum Proteobacteria, recognized for its widespread presence and abundance, plays a crucial role in nitrogen fixation and nutrient cycling (An et al., 2019; Sun et al., 2019). In our findings, Proteobacteria emerged as the predominant phylum within the nosZ-type denitrifying microbial community in the various treatments, with an average relative abundance of 89.54%. This observation aligns with earlier studies that underscore the significant role of this phylum (Zhang et al., 2023; Zhou et al., 2023). *Microvirga* has been identified as the predominant genus of nosZ-denitrifying microorganisms in the Alpine wetland. Recent studies have also demonstrated that *Microvirga* is one of the dominant nosZ-denitrifying microbial groups in surface soils (Fudjoe et al., 2023). A series of previous studies have revealed that *Microvirga* is involved in processes such as nitrogen fixation and antibiotic production, influencing environment-related factors, and plays a significant role in regulating soil N_2_O emissions (Sun et al., 2017; Schmidt et al., 2019).

Notably, changes in precipitation significantly affected the relative abundance of *Azospirillum*, with an inverse relationship observed alongside increased precipitation. This may be attributed to a decline in pH levels due to heightened precipitation, which further reduces the relative abundance of *Azospirillum* (Urovec et al., 2021; Bleken and Rittl, 2022). Meanwhile, the 50% rainfall enhancement treatment employed in this study resulted in a notable increase in soil moisture, corroborating the explanation for the diminished presence of *Azospirillum*, an aerobic nitrogen-fixing bacterium, following increased precipitation (Steenhoudt and Vanderleyden, 2000).

### Factors influencing the diversity and composition of nosZ-type denitrifying microbial communities

The hierarchical analysis revealed that total nitrogen (TN) content was the most influential explanatory variable for community diversity, accounting for 14.9% of the variation observed. This finding is substantiated by a series of previous studies. For instance, Zhang (2023) examined the abundance, community diversity, and environmental drivers of nosZ-type denitrifying bacteria in lake sediments in Inner Mongolia, demonstrating that the diversity of these denitrifying microorganisms containing the *nosZ* functional gene varied in relation to TN content. Zhou et al. (2023) further explored the impact of nitrogen inputs on the community structure of nitrous oxide reductase gene denitrifying bacteria, affirming the close relationship between TN and the nosZ denitrifying microbial community.

Moreover, our study found that environmental factors exerted a negative influence on the structure of the nosZ-type denitrifying microbial community, with pH emerging as the most significant explanatory factor. This observation aligns with the findings of Meng et al. (2019), whose research on the diversity and abundance of denitrifying bacteria during cow manure composting concluded that pH could account for noteworthy differences in the distribution of *nosZ* gene species. Meng et al. (2023) similarly reported that soil pH significantly affected the abundance of denitrifying genes in rice paddies.

Contrastingly, some studies present divergent findings. For example, Yeerken et al. (2024), in their examination of N_2_O-reducing bacteria in river systems, identified dissolved organic carbon (DOC) as a crucial environmental factor influencing *nosZ* gene abundance. Yang et al. (2020) investigated denitrifying bacteria in northeastern black soil, revealing that soil available phosphorus (AP) and soil organic matter (SOM) were the principal determinants of *nosZ* gene abundance. Fudjoe et al. (2023), in their study of semi-arid maize fields, reported that soil organic carbon (SOC), TN, nitrate nitrogen (NO_3_^–^−N), and pH had significant impacts on the composition of nosZ-type denitrifying bacteria. This discrepancy may stem from the fact that high-altitude and low-temperature environments are more conducive to inhibiting nitrogen mineralization, thereby maintaining relatively high levels of TN. Recent research by Zhang et al. (2025) on the vertical differentiation of soil microorganisms during the freezing period of Qinghai Lake provides corroborative evidence, demonstrating that TN content gradually increases with rising elevation gradients.

In summary, these findings highlight the intricate interplay of factors influencing denitrifying microbial communities across varied environments, emphasizing the nuanced roles of precipitation changes and soil characteristics in shaping these communities.

### Construction of the nosZ-type denitrifying microbial community and soil differential metabolites under precipitation changes

In this investigation, the development of the nosZ-type denitrifying microbial community within the various treatments was predominantly governed by deterministic processes associated with heterogeneous selection. Notably, drift effects were observed during the community assembly under the 50% reduced precipitation treatment, accounting for 33.3%. In contrast, the study conducted by Du et al. (2023) on the effects of precipitation on bacterial community diversity and assembly processes in the Gurbantunggut Desert found that dispersal limitation was the primary driver of community assembly in arid conditions. This discrepancy with our findings may be attributed to the inherent water scarcity prevalent in arid environments. Conversely, research by Zhang et al. (2024), which explored denitrifying microorganisms in alpine wetlands, confirmed that heterogeneous selection was the dominant mechanism shaping microbial community assembly in response to precipitation variability, aligning with the results of our study.

Within the treatments, the differential metabolites exhibited significant positive correlations, indicating that changes in precipitation had a profound impact on the relative abundance of metabolites across six identified subgroups. Notably, the relative abundance of fatty acid ester metabolites displayed a trend of initial decline followed by resurgence as precipitation increased, while the relative abundance of metabolites from the remaining four subgroups exhibited an inverse relationship with increasing precipitation. Among these metabolites, N-Acetylaspartic acid, an amino acid integral to nitrogen transformation during its conversion and decomposition, plays a crucial role in sustaining nitrogen balance and bioavailability (Broughton et al., 2015). The correlation analysis also confirmed the positive correlation between N-Acetylaspartic acid and carbon-nitrogen content. N-Acetylaspartic acid was also positively correlated with *Dechloromonas*, which may be attributed to this microbial community’s role as a solid-phase denitrifiers, capable of utilizing lignocellulose as a carbon source, this provides a stable carbon supply for heterotrophic denitrification reactions (Feng et al., 2017; Yang et al., 2021). Syringic acid showed a significant negative correlation with *Dechloromonas*, which is likely closely related to the high antioxidant activity of Syringic acid. Furthermore, soil moisture was significantly negatively correlated with N-(3,4-dimethoxyphenethyl)-N’-(3,4,5-trimethoxybenzoyl) thiourea, aminoimidazole carboxamide ribonucleotide, and Aldosterone. This is generally attributed to increased moisture, which hinders the diffusion of oxygen in the soil, thereby inhibiting microbial metabolic pathways (Albanna et al., 2007).

## Conclusion

In this study, we explored the impacts of precipitation changes on the nosZ-type denitrifying microbial community, soil physicochemical properties, and soil metabolites in the alpine wetland surrounding Qinghai Lake. Notably, *Azospirillum* emerged as the dominant genus, with its abundance significantly influenced by the variations in precipitation. The total nitrogen content stood out as the most critical explanatory factor for the diversity of the microbial community, while pH was identified as the primary determinant of community structure. Moreover, our results highlighted that change in precipitation significantly influenced the relative abundance of N-Acetylaspartic acid. This observation implies that fluctuations in precipitation could have far-reaching consequences on carbon storage within the alpine wetland soil ecosystem of Qinghai Lake, as well as disrupt the existing nitrogen balance within these ecosystems.

In conclusion, our study provides valuable insights into the potential impacts of climate change on wetland ecosystems. Implementing adaptive management strategies will help mitigate the effects of precipitation variability on ecosystem services. However, the research was focused solely on the source wetland in the Tibetan Plateau, and the findings may not be broadly applicable or fully reflective of denitrifying microbial community changes in other regions or wetland types. Further research is needed to better understand the long-term ecological consequences of precipitation changes across different regions and wetland types.

## Data Availability

The datasets presented in this study can be found in online repositories. The names of the repository/repositories and accession number(s) can be found in the article/Supplementary material.

## Footnotes

1
https://www.qiime2.org/

2
ftp://ftp.ncbi.nih.gov/blast/db/

## Supplementary material

Supplementary Figure 1 Sampling point distribution.

Supplementary Figure 2 Rarefaction curve of samples.

Supplementary Figure 3 PCA principal component analysis.

Supplementary Figure 4 Dominant phyla in treatments.

Supplementary Figure 5 PCA principal component analysis and PLS-DA partial least square analysis of metabolites.
