# Supplementary figures and images for: Response of the nosZ-type denitrifying microbial community and metabolic characteristics to precipitation changes in the alpine wetland
Source: Front Microbiol. 2025 Apr 24;16:1581432. doi: 10.3389/fmicb.2025.1581432 (PMC12067595; doi:10.3389/fmicb.2025.1581432)

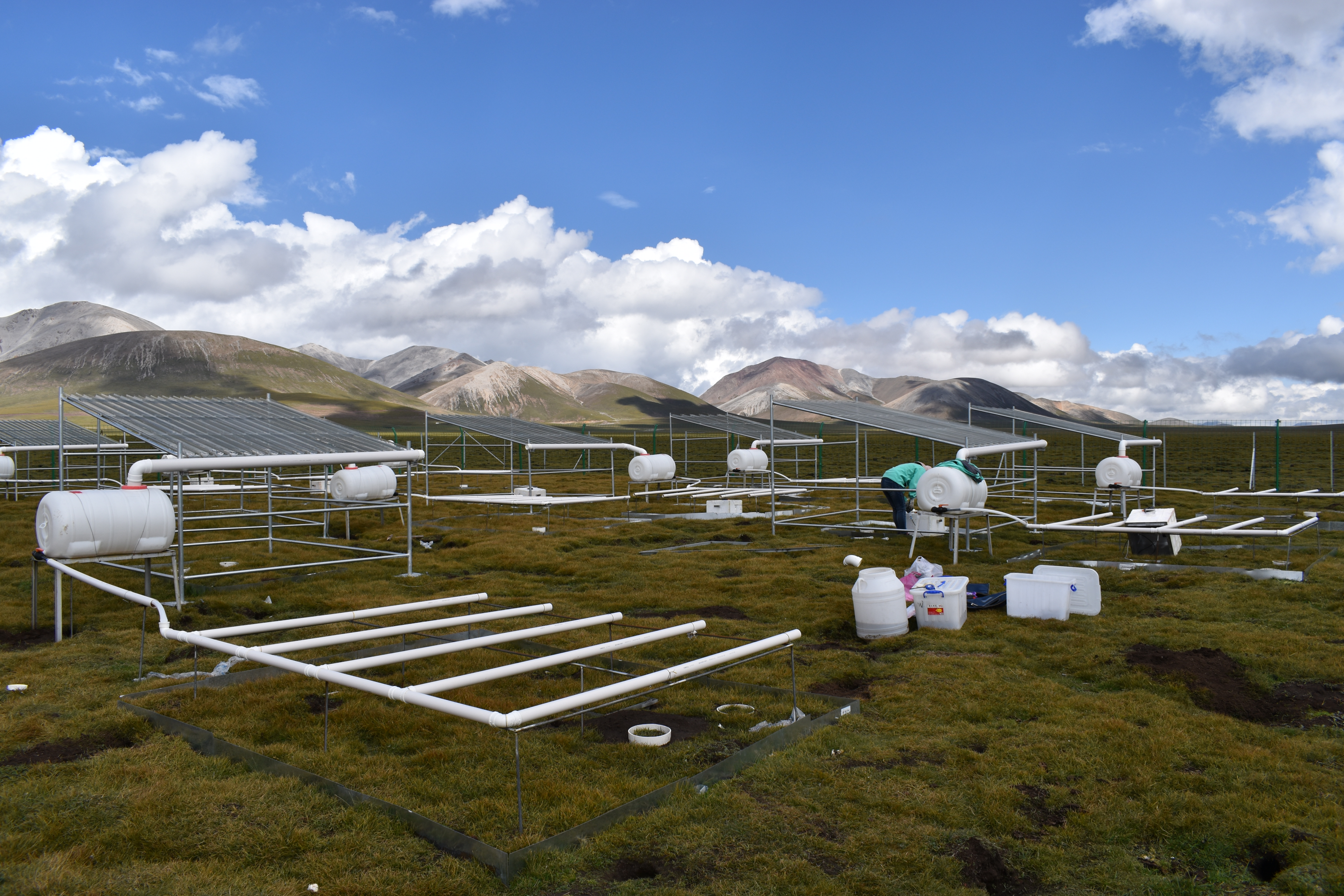

Supplement: Supplementary Figure 1 — Sampling point distribution. [file Data_Sheet_1.pdf]

— -50% — -25% — C — +25% — +50%

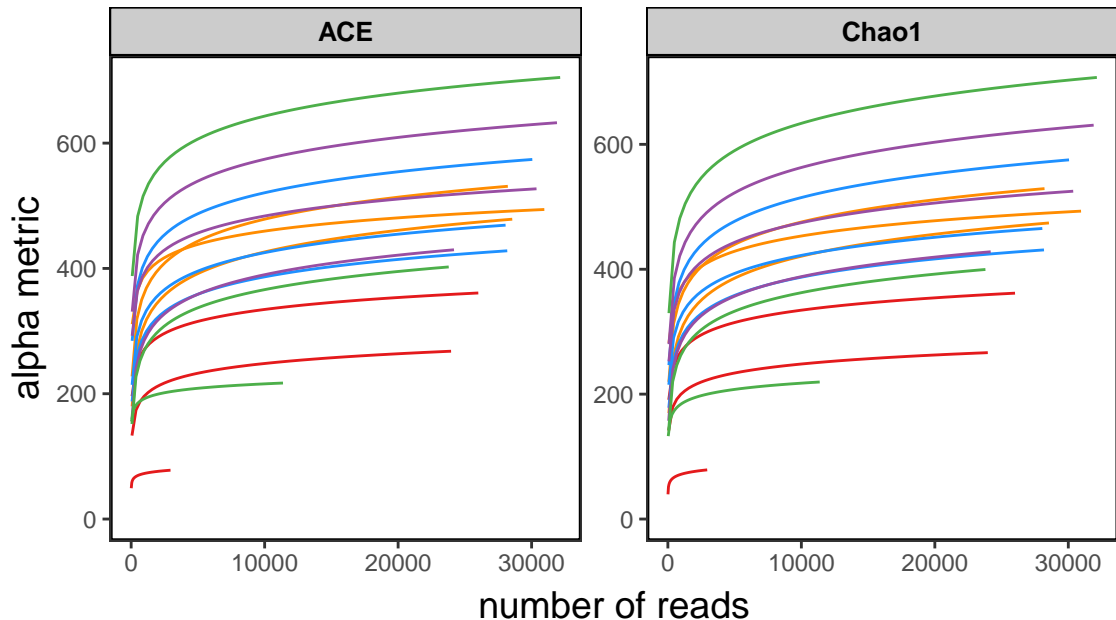

Supplement: Supplementary Figure 2 — Rarefaction curve of samples. [file Data_Sheet_2.pdf]

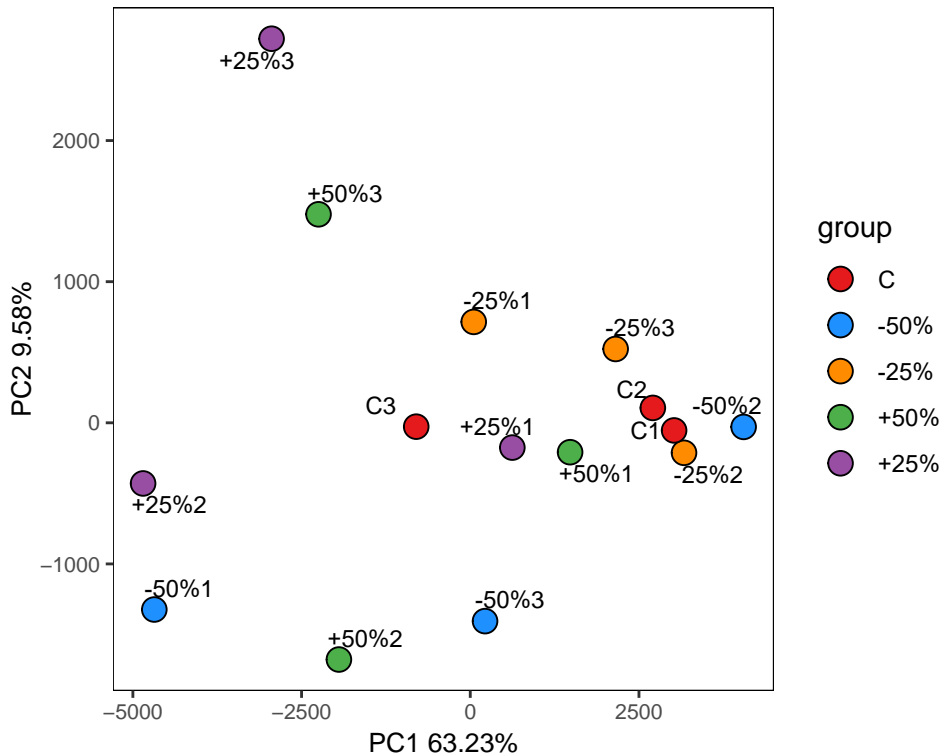

Supplement: Supplementary Figure 3 — PCA principal component analysis. [file Data_Sheet_3.pdf]

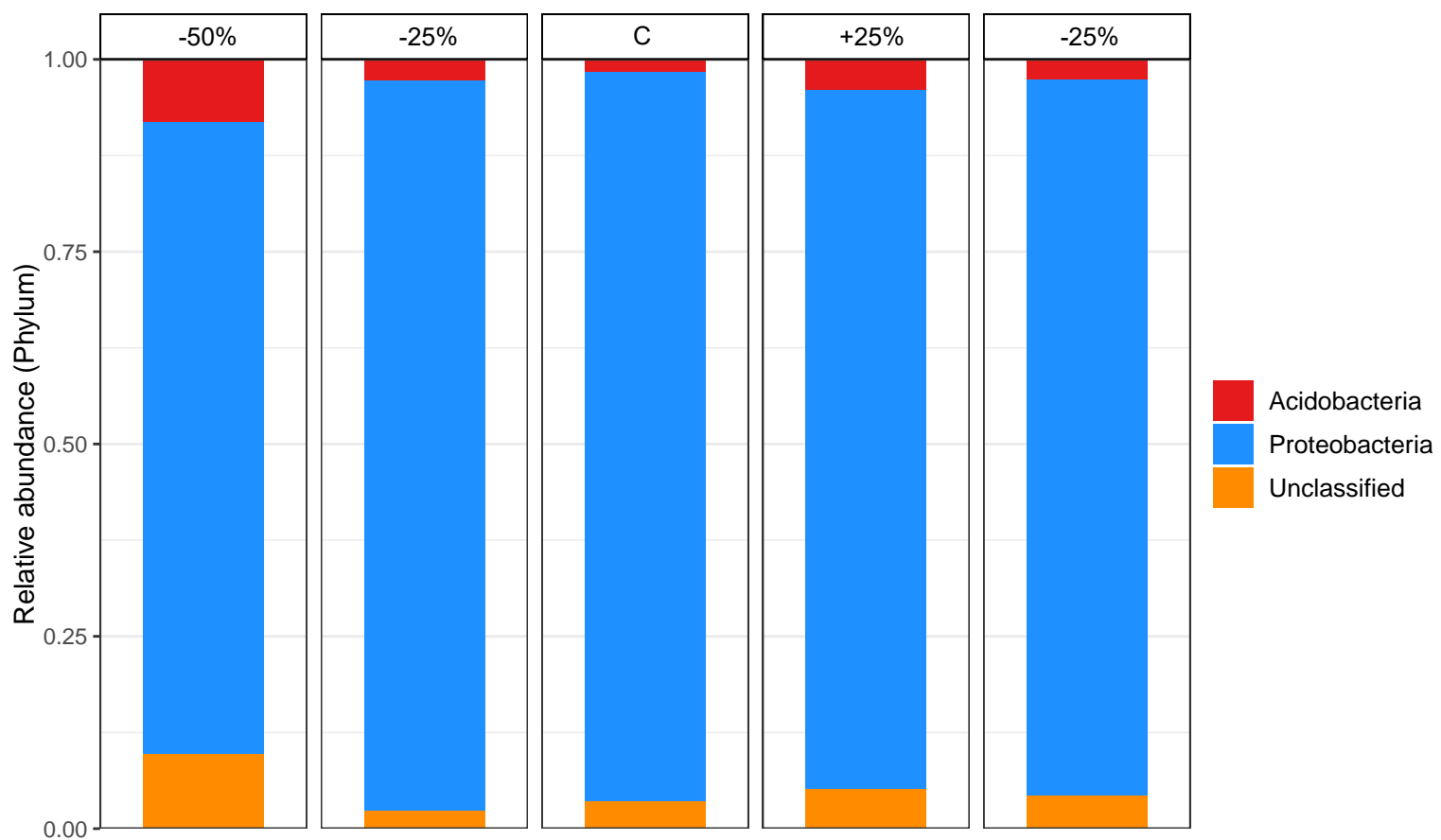

Supplement: Supplementary Figure 4 — Dominant phyla in treatments. [file Data_Sheet_4.pdf]

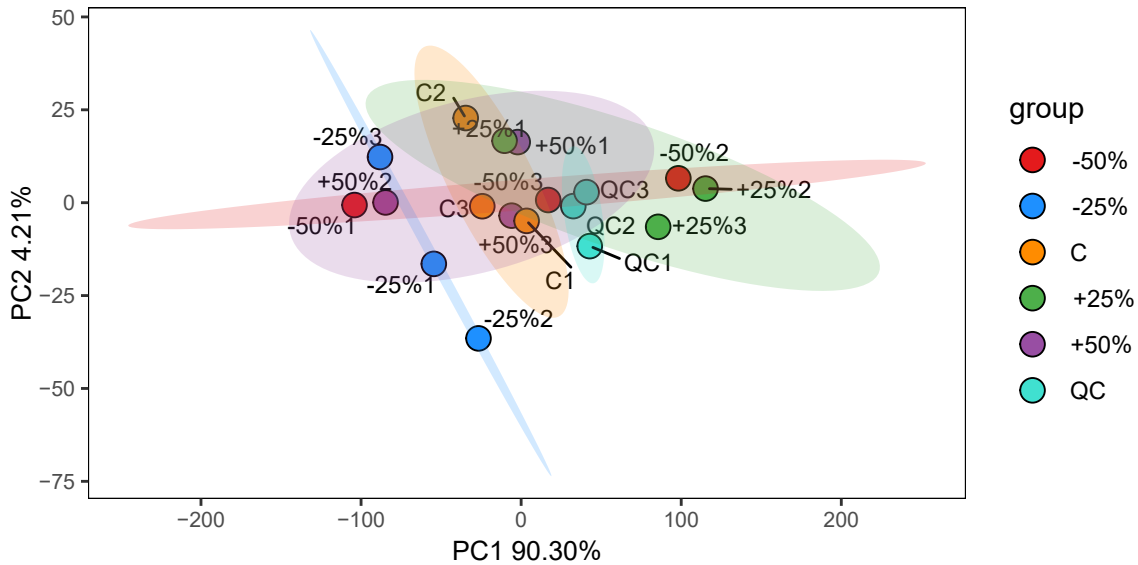

Supplement: Supplementary Figure 5 — PCA principal component analysis and PLS-DA partial least square analysis of metabolites. [file Data_Sheet_5.pdf]
